# Supplementary material for: A lunar core dynamo limited to the Moon’s first ~140 million years
Source: Commun Earth Environ. 2024 Sep 6;5(1):456. doi: 10.1038/s43247-024-01551-z (PMC11379625; doi:10.1038/s43247-024-01551-z)
Supplement: Supplementary file 2 — Supplemental Material [file 43247_2024_1551_MOESM2_ESM.pdf]

Supplementary Information for 'A lunar core dynamo limited to the Moon's first ∼140 million years'

Tinghong Zhou[1], John A. Tarduno[1,2,3*], Rory D. Cottrell[1], Clive R. Neal[4], Francis Nimmo[5], Eric G. Blackman[2,3], Mauricio Ibañez-Mejia[6]

[1]Department of Earth and Environmental Sciences, University of Rochester, Rochester, NY 14627, USA
[2]Department of Physics and Astronomy, University of Rochester, Rochester, NY 14627, USA
[3]Laboratory for Laser Energetics, University of Rochester, Rochester, NY 14623, USA
[4]Department of Civil Engineering and Geological Sciences, University of Notre Dame, Notre Dame, IN 46556, USA
[5]Department of Earth and Planetary Sciences, University of California, Santa Cruz, CA 95064, USA
[6]Department of Geosciences, University of Arizona, Tucson, AZ 85721, USA
[*] e-mail: john.tarduno@rochester.edu

Contents:

- Supplementary Discussion
- Supplementary References
- Supplementary Figures 1-8
- Supplementary Table 1

Supplementary Discussion

Mineral Alteration in Lunar Samples

The formation of lunar magnetic minerals form from the reduction of precursor phases, and as such this is fundamentally different from the formation processes of common magnetic phases in typical terrestrial rocks[1−2]. The generally reduced phases are susceptible to alteration in the laboratory with applied heatings needed for robust paleointensity analyses, involving chemical and/or structural changes[1,3]. A controlled (inert) atmosphere is sometimes used in thermal demagnetization experiments on terrestrial rocks to limit alteration in the laboratory. In some cases this is successful, but in others it leads to reduction and the formation of new magnetic minerals, compromising magnetic data (see discussion in ref 4). The use of a highly reducing atmosphere[5], mimicking that thought to exist on the Moon during the formation of lunar magnetic minerals, is problematic because it could lead to the further reduction of Fe-bearing phases, producing new magnetic minerals, and rendering any magnetic data bearing on the ancient Moon unusable. Moreover, this approach does not address structual changes[3].

An alternative approach is to concentrate on kinetics, and rapid heating/cooling to limit mineral alteration. This is achieved in this and prior studies using CO_2 lasers[6−7]. Ultimately, the effectiveness of this approach must be tested in each experiment, and this is done by means of TRM tests. In this study, there are several levels of tests. First, after the initial heating in an applied field, the sample is reheated to the same temperature in a zero field. If new magnetic minerals had formed, the magnetization should not return to the zero state as magnetic interactions amongst the newly formed phases would tend to leave a residual magnetization. Second, after the heating in an initial field (20 μT in this case) our crystals are reheated to a field twice as strong (40 μT). Magnetic mineral formation is time dependent, and if new magnetic minerals are being formed by the applied laboratory heatings we would expect that the magnetization after the second heating should be much larger than expected, corresponding to a magnetization efficiency >>100%. These values are not observed in the crystals studied here, or in prior work[1]. Third, the effects of alteration are variable depending on initial Fe-beating phase content (chemistry and amount), so we require replication for all studies. The SCP results reported here and in prior studies[1] have replicates. Therefore, claims in ref 5 that the lunar SCP values might reflect alteration are erroneous because tests and data specifically exclude alteration.

Debates on Impact Magnetization in Lunar Samples

Impacts can create large magnetic fields through charge separation, a well-established process documented in laboratory experiments[8] and studied through modeling of asteroid[9] and cometary impacts[10]. There are now three Apollo samples that provide evidence for magnetization from an impact plasma: Apollo 70019, 15498, and 64455. Studies of 70019[11] impact glass lacked pTRM checks,

but the regular linear decay of remanence with increasing demagnetization alone is strong evidence for an impact magnetization. Magnetizations from Apollo breccia 15498 were originally reported by Gose et al.[12] who interpreted these as of internal origin. Glass from the sample was studied by Tikoo et al.[13] who also called for a core dynamo, but this interpretation is contradicted by the new paleointensity data as well as unmagnetized parts of the sample. Tarduno et al.[1] highlighted that the 15498 glass data that yield high unblocking temperature magnetizations record essentially null fields at intermediate to low temperatures (250 to 540 ^{o}C) and are more compatible with the presence and then absence of a magnetizing field, as might happen if the 15498 passed through an impact plasma, or that plasma dissapated. This interpretation is also consistent with the unmagnetized glass of 15498. Arguably the best impact magnetization recorder is Apollo 64455 because this ballistic-shaped glass-coated sample[14−15] most likely traveled through the impact plasma that created South Ray crater. Thermal and nonthermal techniques yield paleointensities that match independent predictions from impact modeling[1].

Proponents of a lunar dynamo have attempted to criticize the 64455 data in several ways. We note that impact magnetization of 64455 is not required for our explanation of our WRP values because this is supported by the independent work on impact plasma magnetization[8−10]. Nevertheless we address the critiques here for completeness, and to further illustrate how the complexities of Apollo magnetic data can lead to erroneous conclusions. The following comments were made in several coordinated abstracts presented at the 2023 LPSC conference. One claim is that the signal might be due to alteration[5], another that the signal could be due to terrestrial contamination in other laboratories[5,16], another is that there no impact magnetization based on studies of other Apollo glasses and other samples from 64455[16−17], and finally it was claimed that cooling rates in glass are too long to record impact fields[5]. We explain below why the 64455 data contradict each claim.

Alteration can occur with short-duration heating, and that is why pTRM checks are essential. These checks exclude alteration in the 64455 specimens that pass reliability criteria[1]. Twelve percent of the 64455 glass specimens examined in Tarduno et al.[1] passed. This value is typical of terrestrial results[18] but exceedingly high for lunar samples. The experimental alteration checks show that the suggestion of alteration (ref 5) is incorrect.

The 64455 sample studied in Tarduno et al.[1] was a return sample meaning that it had been previously supplied to a different scientist. However, the splits (specimens) used from the sample used by Tarduno et al.[1] can not be the same as in prior studies. That is Leich et al.[19] noted that they studied glass separated from the ground mass ("chipping the sample from the rock"[19]), whereas the glass studied in Tarduno et al.[1] was attached, and subsequently subsampled into 1 mm-sized pieces used for analysis. Moreover, a typical lab contamination signal consists of a single coherent vector (overprint) affecting the entire sample. Tarduno et al.[1] described different behavior in specimens. Specifically "...evidence for multiple components and/or changing directions after field-off thermal treatments" was seen in samples rejected for paleointensity determination. This differential behav-

ior, reported in more detail in Cottrell et al.[20], represents an internal test that reveals the claim of terrestrial contamination (claim of refs 5 and 16) to be spurious.

Apollo 64455 is different from other "splash" glasses of the Apollo 17 collection, and therefore it would not be surprising if some of these cooled after the impact plasma associated with South Ray crater dissapated. However, the data of ref 16-17 are obtained with alternating fields are highly scattered and appear to be dominated by GRM magnetizations, exacerbated by a very large number of AF steps. Because of this methodological limitation, we conclude that these data are of insufficient number, quality and technique (e.g., high quality thermal data are needed) to support conclusions on the presence/absence of lunar impact fields. Moreover, we note that the 64455 magnetization reported in Tarduno et al.[1] has recently been reproduced by independent measurements[21].

Tarduno et al.[1] explained that simple conductive cooling models likely do not yield accurate measures of glass quenching time, and See et al.[22] emphasized that condensation nucleate within the glass could greatly affect prior calculations and more generally that cooling could have been on second timescales. We further note that the great variety of FeNi textures preserved in 64455 glass[1] demand rapid quenching, but it is certainly possible, if not likely, that some glass specimens might have magnetized particles and others might not, because of a hetergenous distribution of the inclusions and the relatively rapid decay of the impact plasma magnetization. Finally, we note that the lifetime of the magnetizing field quoted in ref 5 is erroneous because it does not account for the decay of the field (i.e., an impact plasma field will be present for much longer than the peak field values available from refs. 8-10).

Supplementary References

1. Tarduno, J. A., Cottrell, R. D., Lawrence, K., Bono, R. K., Huang, W., Johnson, C. L., Blackman, E. G., Smirnov, A. V., Nakajima, M., Neal, C. R., Zhou, T., Ibanez-Mejia, M., Oda, H. & Crummins, B. Absence of a long-lived lunar paleomagnetosphere. *Sci. Adv.*, **7**, eabi7647 (2021).
2. Papike, J., Taylor, L. & Simon, S. Lunar minerals, in Lunar Source Book, Heiken, G.H., Vaniman, D.T., French, B.M. Eds. (Cambrige Univ. Press, 1991), chap. 5, pp. 137–153.
3. Dunlop, D. J. & Özdemir, Ö. *Rock magnetism: Fundamentals and Frontiers* (Cambridge Univ. Press, 2001).
4. Dare, M. S., Tarduno, J. A., Bono, R. K., Cottrell, R. D., Beard, J. S., & Kodama, K. P. Detrital magnetite and chromite in Jack Hills quartzite cobbles: Further evidence for the preservation of primary magnetizations and new insights into sediment provenance. *Earth Planet. Sci. Lett.* **451**, 298-314 (2016).
5. Weiss, B. P., Wiecorek, M. A., Gattacceca, J., Tikoo, S. M., McDonald, C., Hodges, K. V., & Lepaulard, C. Evidence for a long-lived lunar dynamo from magnetization in Apollo samples and the lunar crust. *Lunar Planet Sci. Conf.*, 2806 (2023).

6. Tarduno, J. A., Cottrell, R. D., Watkeys, M. K. & Bauch, D. Geomagnetic field strength 3.2 billion years ago recorded by single silicate crystals. *Nature* **446**, 657–660 (2007).
7. O'Brien, T., Tarduno, J. A., Anand, A., Smirnov, A. V., Blackman, E. G., Carroll-Nellenback, J. & Krot, A. N. Arrival and magnetization of carbonaceous chondrites in the asteroid belt before 4562 million years ago. *Commun. Earth Environ.* **1**, 54 (2020).
8. Crawford, D. A., & Schultz, P. H. Laboratory observations of impact-generated magnetic fields. *Nature* **336**, 50-52 (1988).
9. Crawford, D. A., Simulations of magnetic fields produced by asteroid impact: Possible implications for planetary paleomagnetism. *Int. J. Impact Eng.* **137**, 103464 (2020).
10. Bruck Syal, M., & Schultz, P. H., Cometary impact effects at the Moon: Implications for lunar swirl formation. *Icarus* **257**, 194-206 (2015).
11. Sugiura, N., Wu, Y. M., Strangway, D. W., Pearce, G. W., & Taylor, L. A., A new magnetic paleointensity value for a 'young lunar glass'. *Proc. Lunar Planet. Sci. Conf.* **10**, 2189-2197 (1979).
12. Gose, W. A., Strangway, D. W., & Pearce, G. W., A determination of the intensity of the ancient lunar magnetic field. *The Moon* **7**, 196-201 (1973).
13. Tikoo, S. M., Weiss, B. P., Shuster, D. L., Suavet, C., Wang, H., & Grove, T. L., A two-billion-year history for the lunar dynamo. *Sci. Adv.* **3**, e1700207 (2017).
14. Ryder, G. & Norman, M. D. Catalog of Apollo 16 rocks: Part 2 63335-66095 (Curatorial Branch Publication 52, NASA JSC 16904, 1980).
15. A. G. Sanchez, D4. Geology of Stone Mountain, in Geology of the Apollo 16 Area, Central Lunar Highlands, in Geological Survey Professional Paper 1048, G. E. Ulrich, C. A. Hodges, W. R. Muehlbergerm, Eds. (U.S. Gov. Print. Office, 1981), pp. 106–126.
16. Chaffee, T., Tikoo, S. M., Abubo, R., Boeschen, S. G., & Weiss, B. P., Testing whether lunar melt glasses preserve records of impact-generated magnetic fields. LPI Contributions 2806 (2023): 1741.
17. Chaffee, T., Tikoo, S. M., Boeschen, S. G., Abubo, R., Jung, J.-I. & Weiss, B. P., No evidence of magnetization from impact-generated fields in 2 Ma lunar impact melt glasses. GP24A-08, to be presented at AGU23, 11-15 December (2023).
18. Bono, R. K., Tarduno, J. A., Nimmo, F., & Cottrell, R. D. Young inner core inferred from Ediacaran ultra-low geomagnetic field intensity. *Nat. Geosci.* **12**, 143–147 (2019).
19. Leich D.A., Tombrello T.A. & Burnett D.S. The depth distribution of hydrogen and fluorine in lunar samples. *Proc. 4th Lunar Sci. Conf.* 1597-1612 (1973).
20. Cottrell, R. D., Zhou, T., & Tarduno, J.A., Dataset of replicate Apollo sample magnetizations bearing on impacts and absence of a long-lived lunar dynamo *in review.*
21. Tarduno, J. A., Sethuraj K .R., Cottrell, R. D., Vamivakas, N. & Oda H. Quantum diamond microscopy magnetometry without applied fields to reveal the unbiased carriers of natural remanent magnetization, GP41-0499, to be presented at AGU2023, 11-15 December (2023).
22. See, T. H., Hörz, F., & Morris, R. V. Apollo 16 impact-melt splashes: Petrography and major-element composition. *Jour. Geophys. Res.* **91**, E3-E20 (1986).

Supplementary Figure 1. Recalculated Lunar ages using revised decay constants for Lunar samples. 70035 (**a, b**), 75035 (**c**), 14053 (**d**), 12021 (**e, f**), and 12040 (**g, h**). See Supplementary Table 1 for references.

Supplementary Figure 2. Additional silicate crystal TRM experiments for 70035, 75035. **a**, Transmission light photo of 70035 crystal measured. **b**, TRM experiment on the crystal imaged in (a). Intensity versus experiment steps. Efficiency is calculated from the field-on intensities of applied fields of 20 μT and 40 μT. **c**, Transmission light photo of 75035 crystal measured. **d**, TRM experiment as in (b) above.

Supplementary Figure 3. Scanning electron microscope images for subsamples of 70035 feldspar crystal. **a**, SEM backscatter image (20 keV) from 70035,4g1 with analysis spots highlighted by number. **b,c,d**, Analyses at spots 1, 2 and 3, respectively in (a). **e**, Additional SEM backscatter image (20 keV) from 70035,4g1 with analysis spots. **f,g**, Analyses at spots 1, and 2 in (e). **h**, SEM backscatter image (20 keV) from 70035,4g2 with analysis spots. **i,j,k**, Analyses at spots 1, 2, and 3, respectively, in (h). **l**, Additional SEM backscatter image (20keV) from 70035,4g2 with analysis spots. **m**, Analyses at spots 1.

Supplementary Figure 4. Scanning electron microscope images for subsamples of 75035 feldspar crystal. **a**, SEM backscatter image (20 keV) from 75035,49g2, with analysis spots highlighted by number. **b,c,d**, Analyses at spots 1, 2 and 3, respectively, in (a). **e**, Additional SEM backscatter image (20 keV) from 75035,49g2, with analysis spots highlighted by number. **f,g**, Analyses at spots 1 and 2, respectively, in (e). **h**, Additional SEM backscatter image (20 keV) from 75035,49g2, with analysis spot highlighted by number. **i**, Analyses at spots 1 in (h).

Supplementary Figure 5. REM' experiment for whole rock subsample from Apollo samples 70035. **a**, Orthogonal vector plot of AF demagnetization of NRM for 70035,4d (inset picture). The data presented are after two successive 3-point moving averages (Methods). Demagnetization steps labeled in gray. Blue circles, horizontal projection; red squares, vertical projection. **b**, Orthogonal vector plot of AF demagnetization of a saturation remanent magnetization after 2 successive steps of 3-point moving average smoothing. Symbols as in (a). **c**, NRM versus saturation remanent magnetization (circles). **d**, Expansion of orthogonal vector plot from 90-160 mT in a. Green arrows, vector corresponding to demagnetization range used in the diectional fit. **e**, Expansion of orthogonal vector plot from 90-160 mT in b. **f**, Expansion of NRM versus saturation remanent magnetization from 90-160 mT in c. Demagnetization range corresponding to directional fit shown in blue.

Supplementary Figure 6. Additional silicate crystal TRM experiments for 61016, 60025. **a**, Transmission light photo of 61016 crystal measured. **b**, TRM experiment on the crystal imaged in (a). Intensity versus experiment steps. Efficiency is calculated from the field-on intensi-

ties of applied fields of 20 μT and 40 μT. **c**, Transmission light photo of 60025 crystal measured. **d**, TRM experiment as in (b) above.

Supplementary Figure 7. Scanning electron microscope images for subsamples of 61016 feldspar crystal. **a**, SEM backscatter image (15 keV) from 61016,177g2, with analysis spots highlighted by number. **b,c,** Analyses at spots 1 and 2, respectively, in (a). **d**, SEM backscatter image (15 keV) from 61016,177g3,with analysis spots highlighted by number. **e,f**, Analyses at spots 1 and 2 in (d). **g**, SEM backscatter image (15 keV) from 61016,177g3 presented in Figure 5 (e) with analysis spots highlighted by number. **j**, Additional SEM backscatter image (15 keV) from 61016,177g3, with analysis spot highlighted by number. **k,l,m,n**, Analyses at spots 1, 2, 3 and 4, respectively, in (j).

Supplementary Figure 8. Scanning electron microscope images for subsamples of 60025 feldspar crystal. **a**, SEM backscatter image (20 keV) from 60025,869g2, with analysis spots highlighted by number. **b,c,d**, Analyses at spots 1, 2 and 3, respectively, in (a). **e**, SEM backscatter image (20 keV) from 60025,869g3, with analysis spots highlighted by number. **f,g**, Analyses at spots 1, and 2, respectively in (e). **h**, Additional SEM backscatter image (20 keV) from 60025,869g3, with analysis spots highlighted by number. **i,j,k**, Analyses at spots 1, 2 and 3 respectively in (h). **l**, Additional SEM backscatter image (20 keV) from 60025,869g3, with analysis spot highlighted by number. **m**, Analyses at spots 1 in (l).

Supplementary Table 1. Recalibrated ages for Apollo samples yielding SCP data.

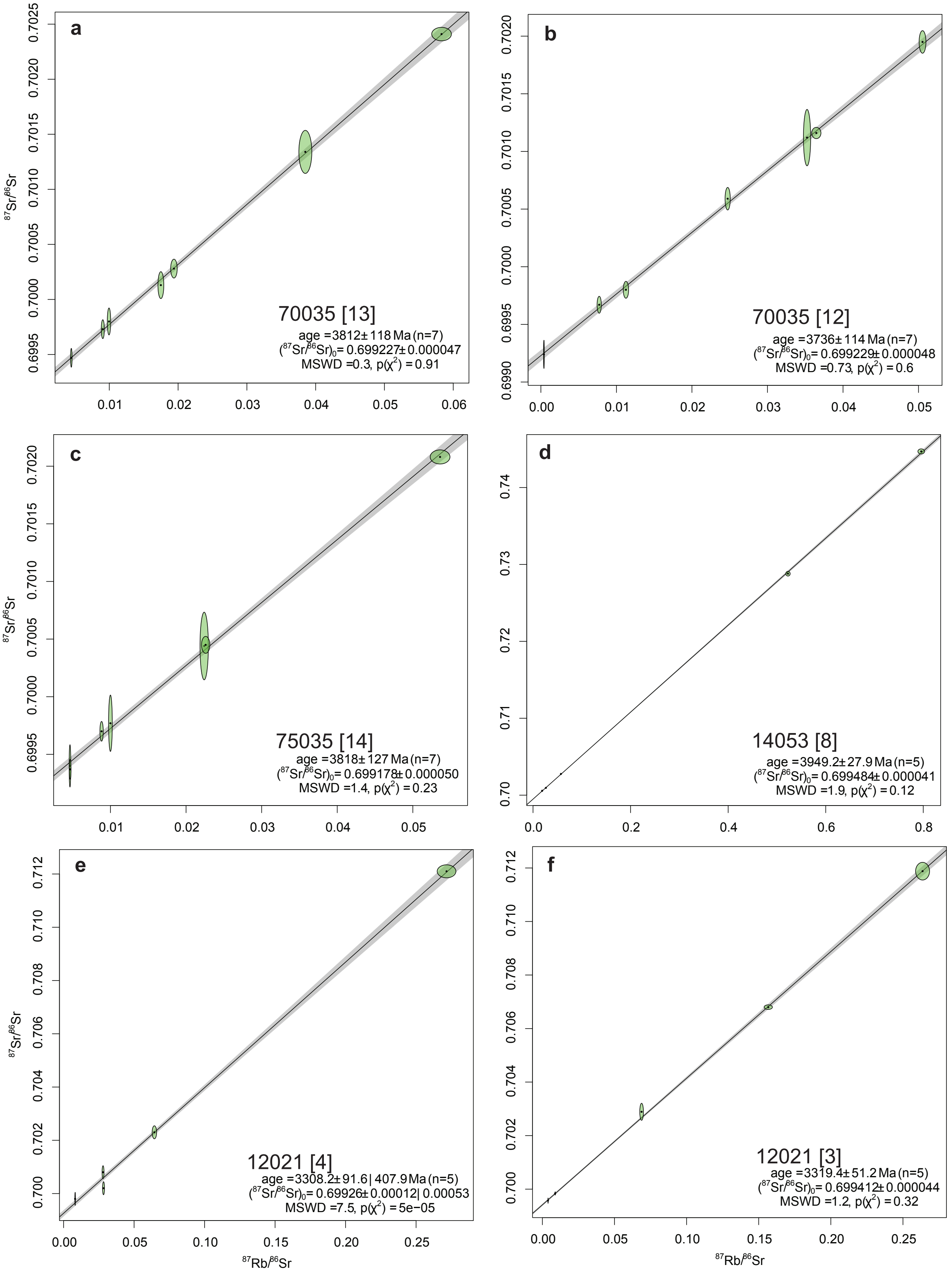

Supplementary Figure 1

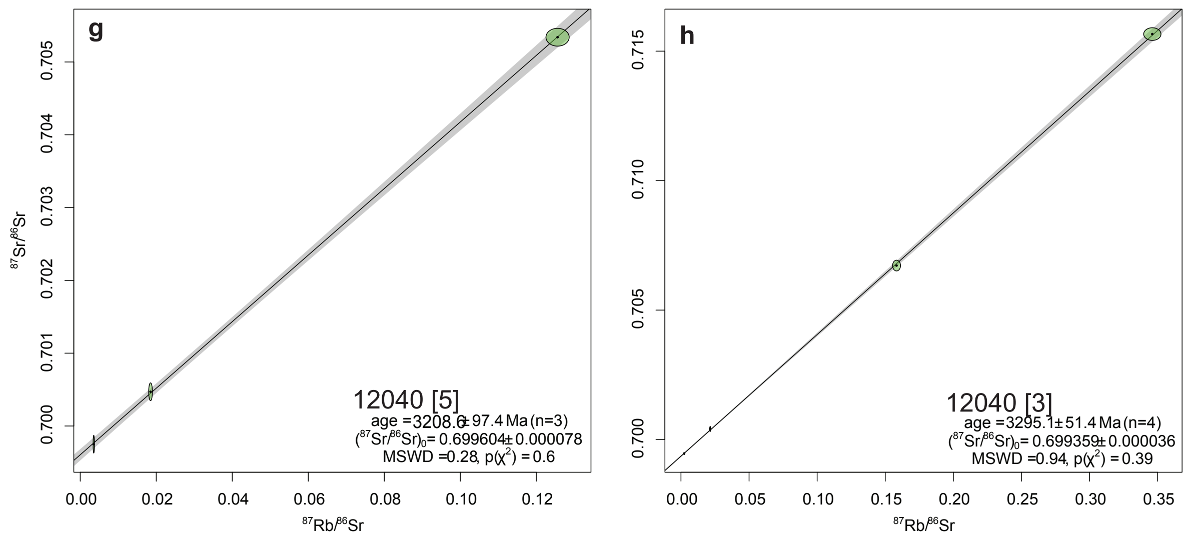

Supplementary Figure 1 continued

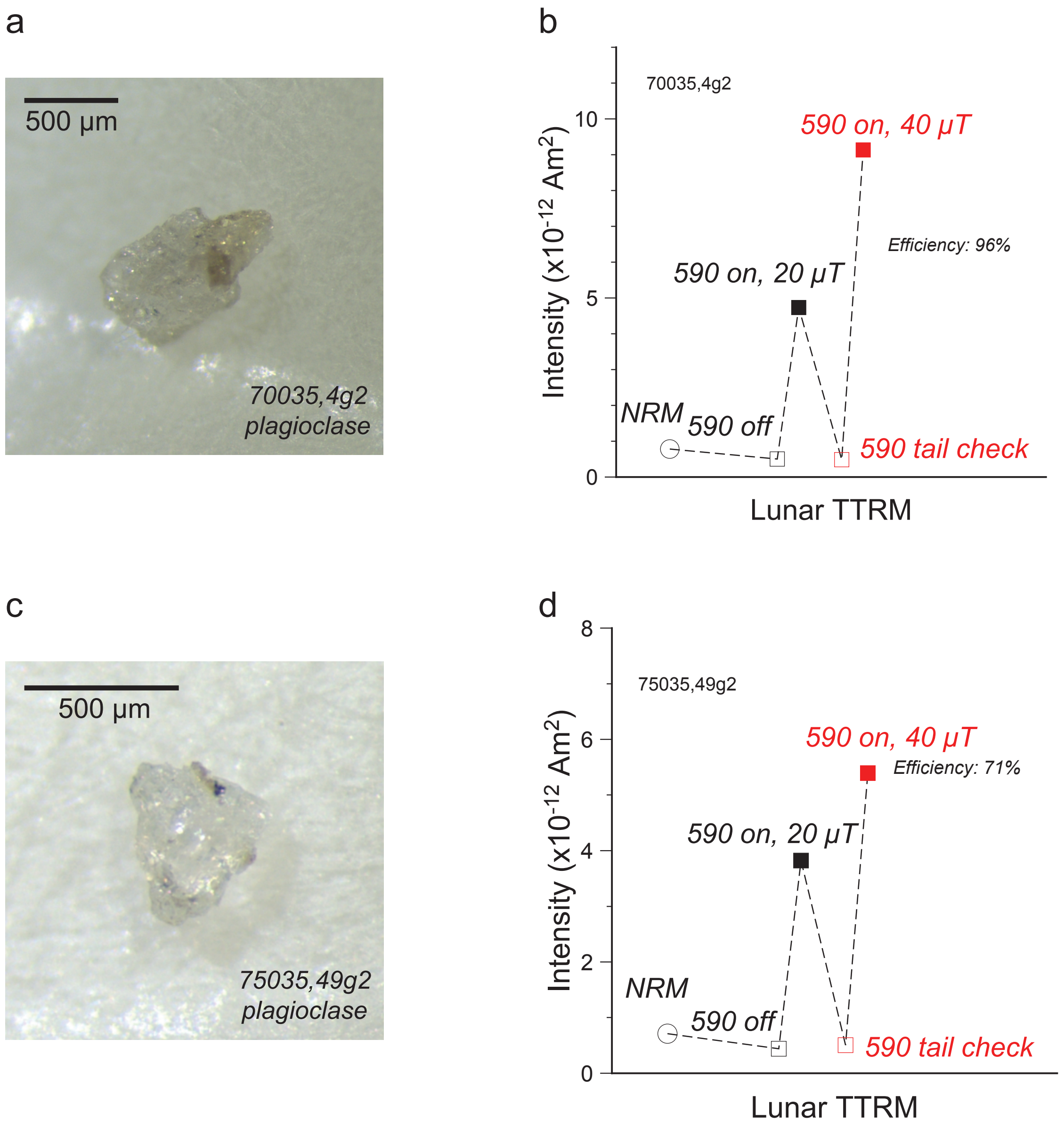

Supplementary Figure 2

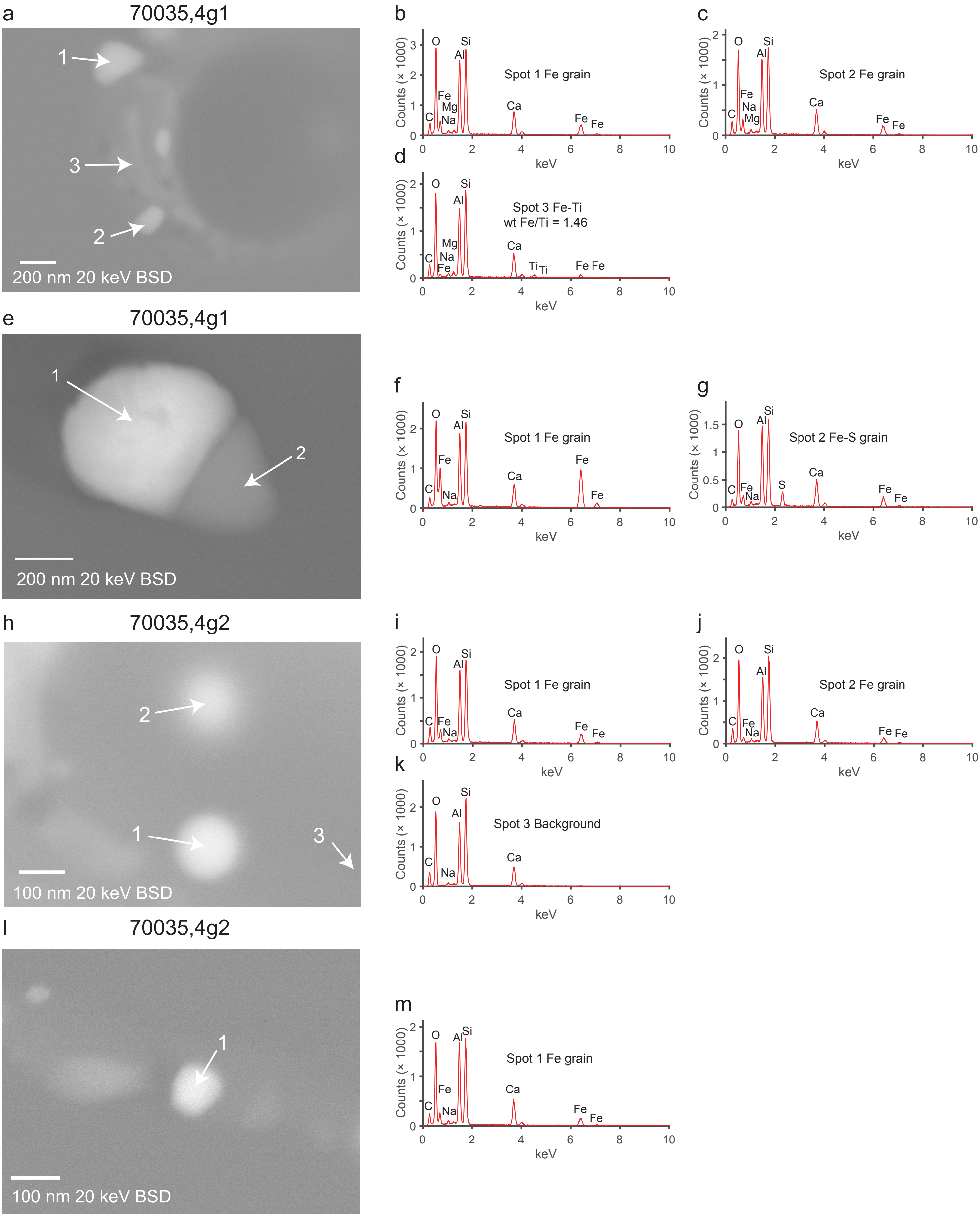

Supplementary Figure 3

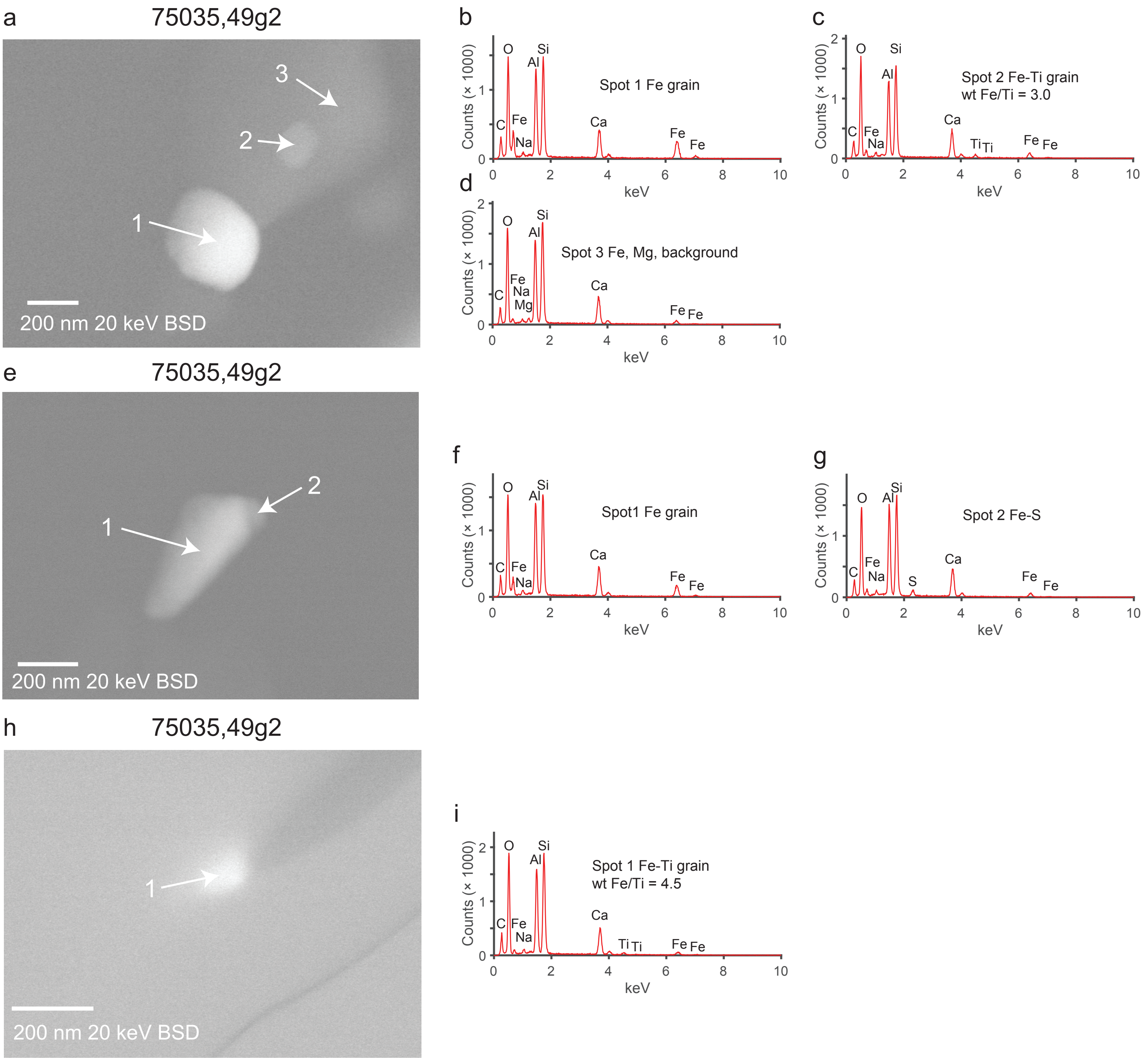

Supplementary Figure 4

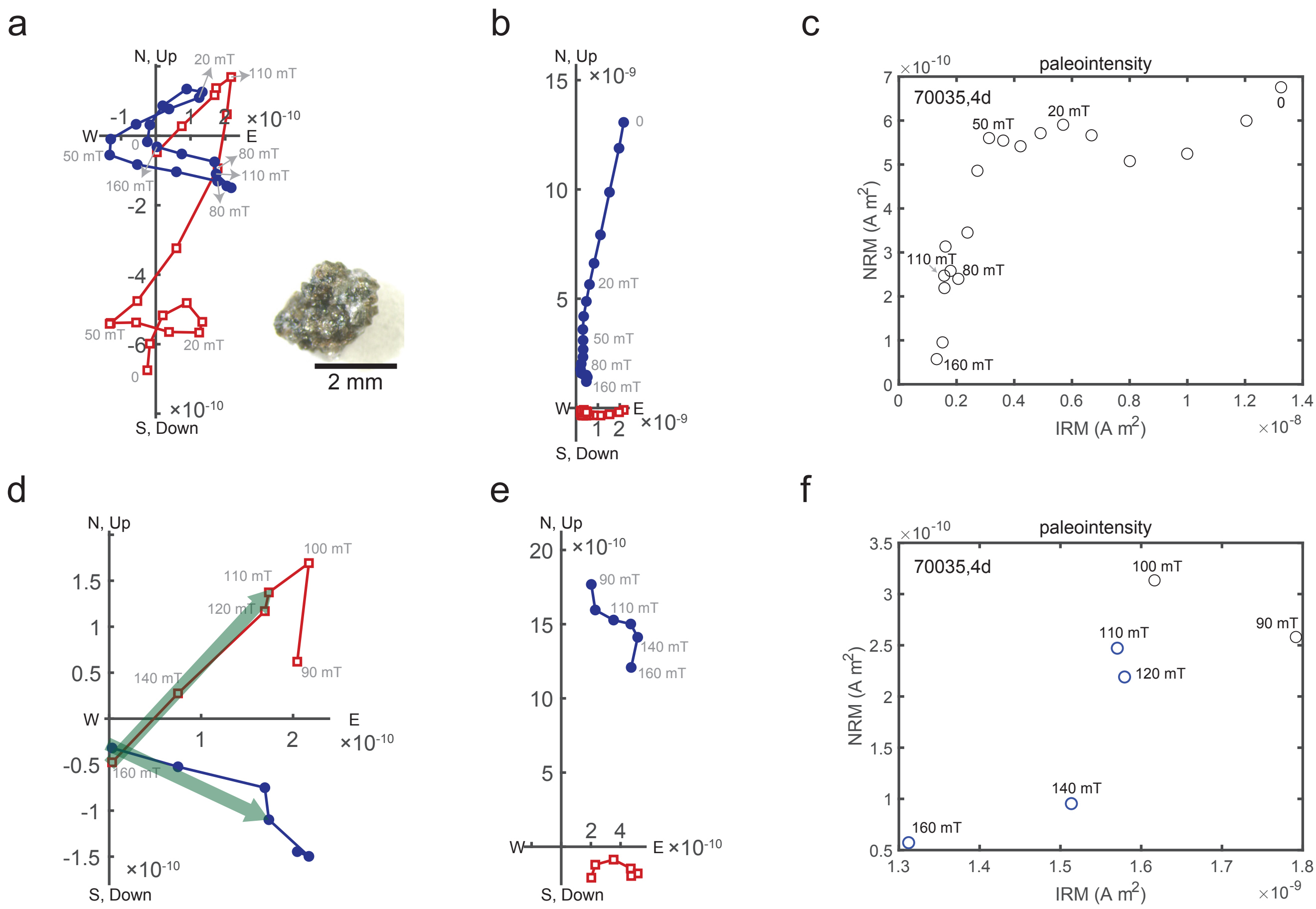

Supplementary Figure 5

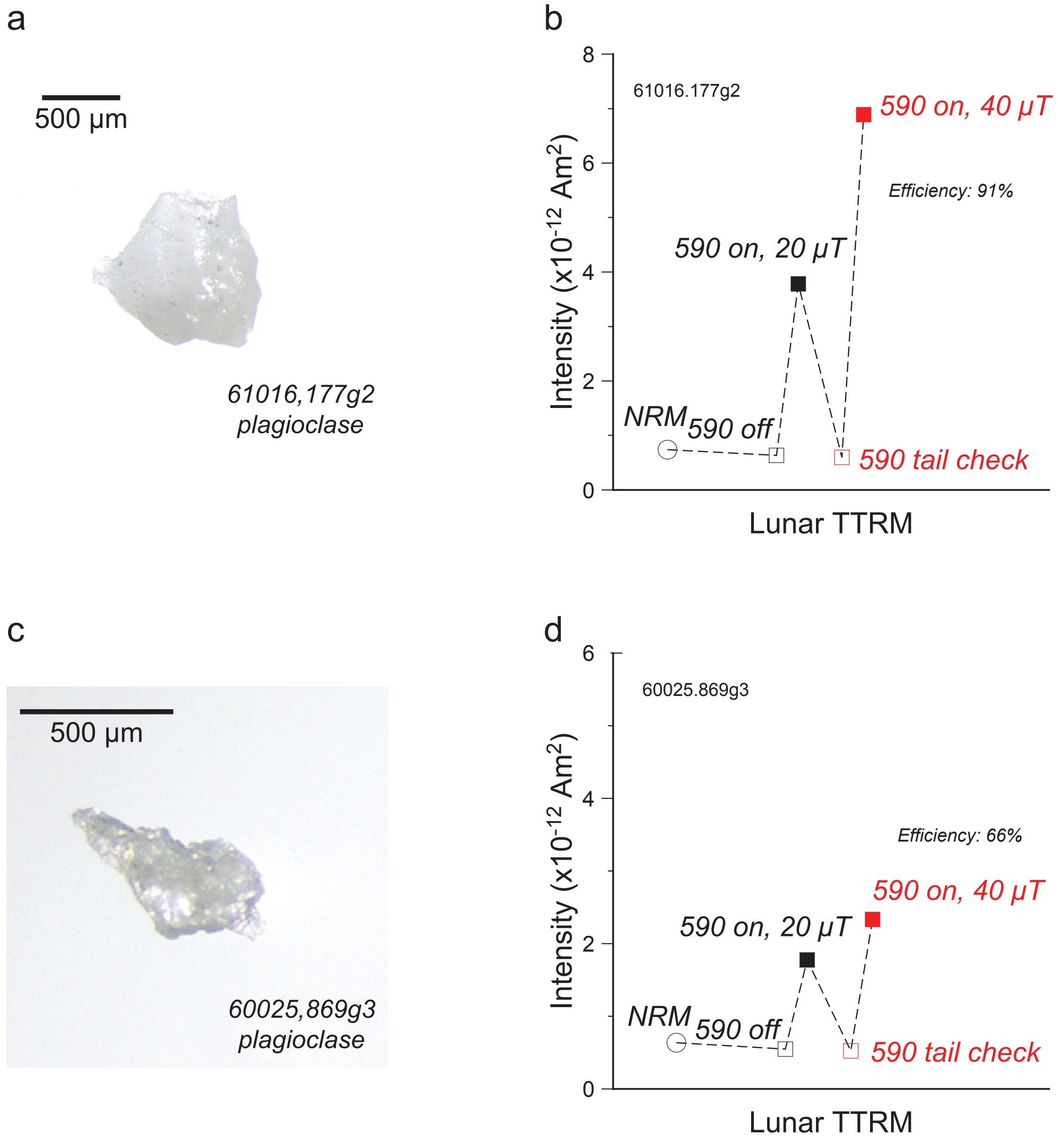

Supplementary Figure 6

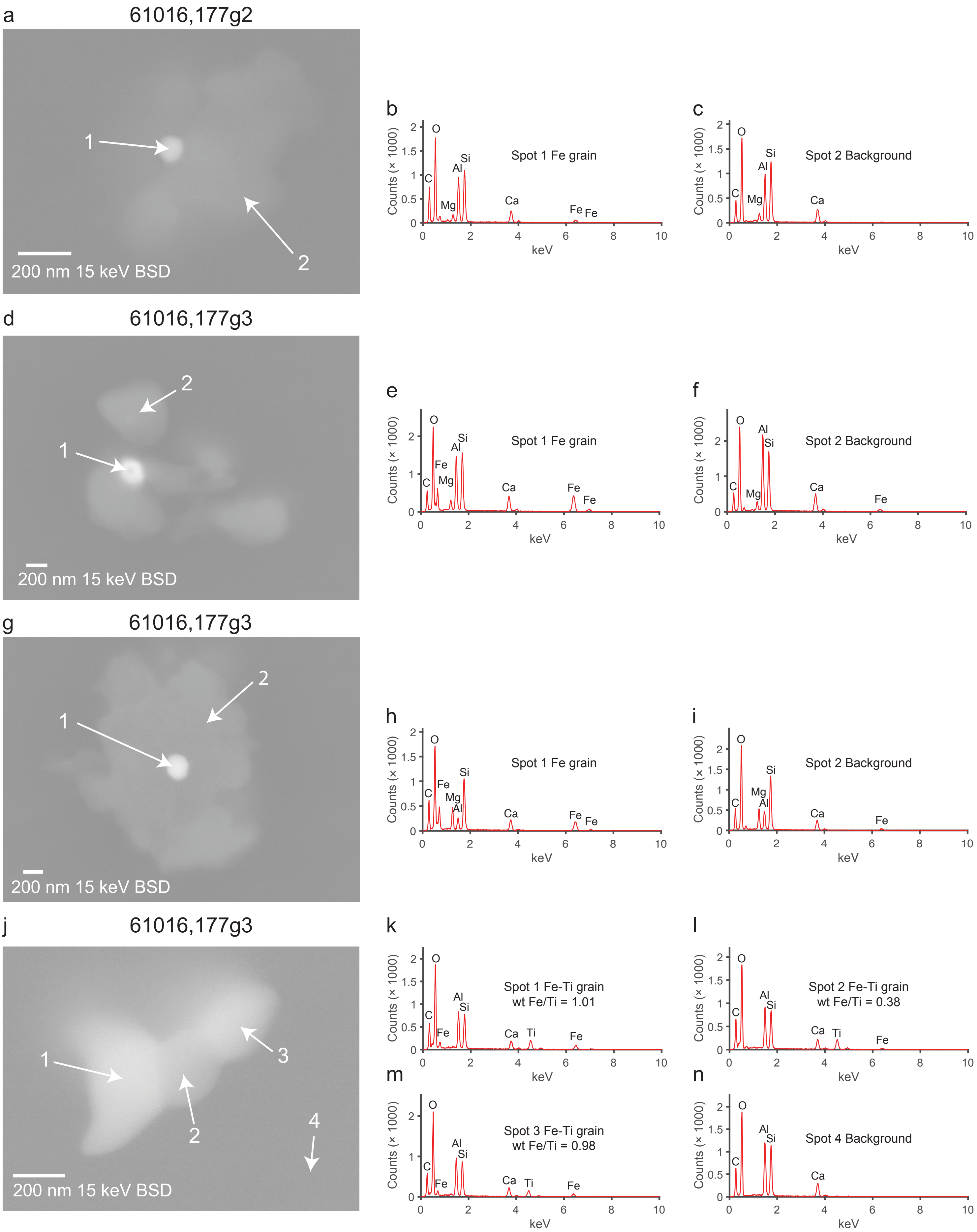

Supplementary Figure 7

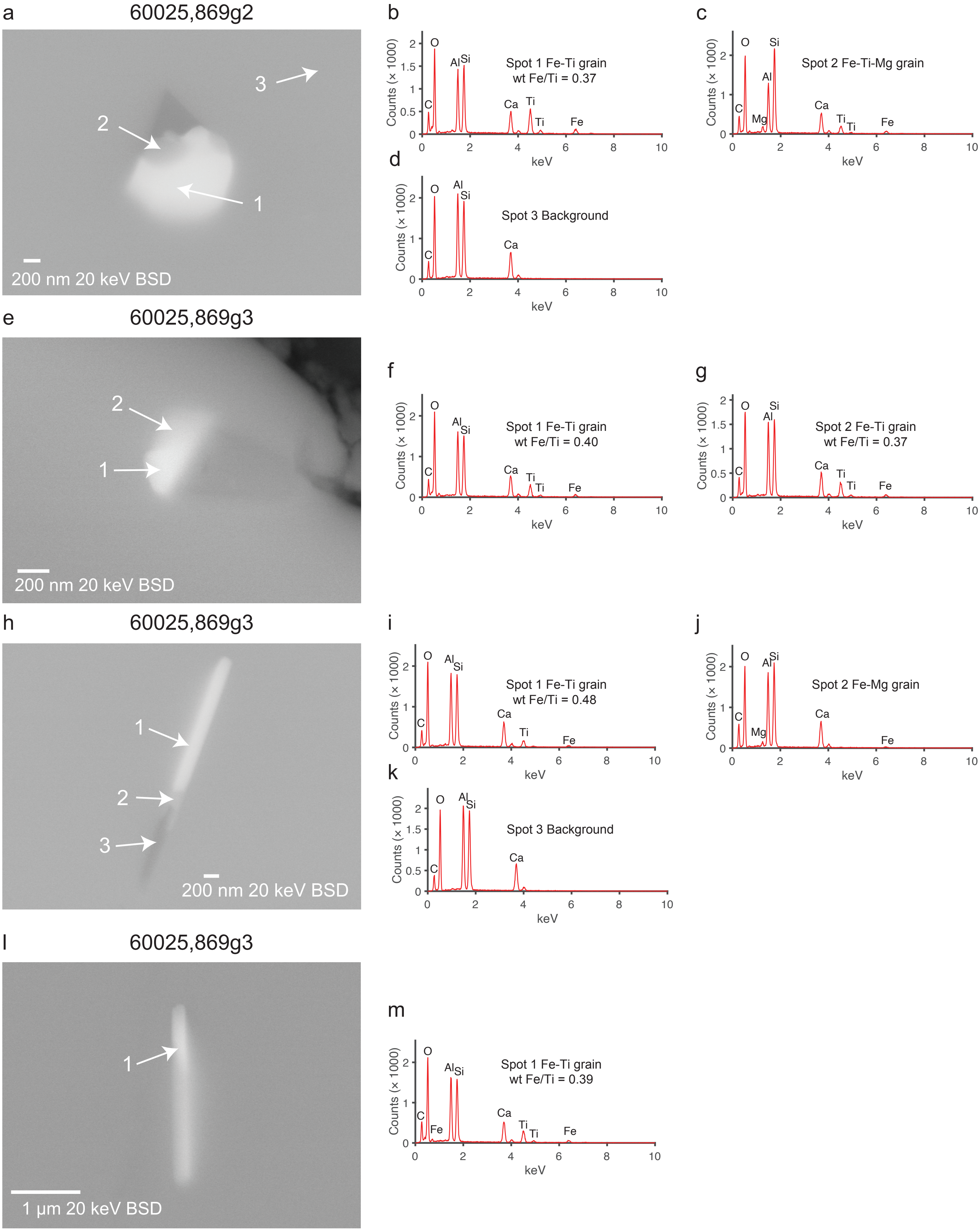

Supplementary Figure 8

Supplementary Table 1: Lunar sample age recalculations

Sample	System	Recal-culated	Age (Ma)	±2σ	MSWD	Decay constant (if recalc.)	Data Ref.	Notes
**12021**	Rb-Sr	x	3319	51	1.2	[1]	[3]	
	Rb-Sr	x	3308	92	7.5	[1]	[4]	
Mean			*3316*	*44*				
**12040**	Rb-Sr	x	3295	51	0.94	[1]	[3]	
	Rb-Sr	x	3208	97	0.28	[1]	[5]	
Mean			*3276*	*44*				
**12053**	39Ar/40Ar		3170	60	NA	NA	[6]	Not recalculated; insufficient published information
	U-136Xe		3150	600	NA		[7]	
Mean			*3170*	*59*				
**14053**	Rb-Sr	x	3949	28	1.2	[1]	[8]	
	39Ar/40Ar	x	3857	50	2	[2]	[9]	Final age uncertainty includes quadratic propagation of analytical and flux monitor uncertainties.
	39Ar/40Ar	x	3871	47	2	[2]	[9]	Final age uncertainty includes quadratic propagation of analytical and flux monitor uncertainties.
Mean			*3915*	*21*				
**60025**	Pb-Pb		4359.2	2.4	1.6		[10]	
	147Sm-143Nd		4367	11	0.4		[10]	
	146Sm-142Nd		4318	+30/-38			[10]	
Mean			*4360*	*3*			[10]	
**61016**	U-136Xe		3970	250			[11]	
**70035**	Rb-Sr	x	3736	114	0.73	[1]	[12]	
	Rb-Sr	x	3812	118	0.3	[1]	[13]	
	39Ar/40Ar	x	3656	60	1.2	[2]	[9]	Final age uncertainty includes quadratic propagation of analytical and flux monitor uncertainties.
	39Ar/40Ar	x	3686	51	1.7	[2]	[9]	Final age uncertainty includes quadratic propagation of analytical and flux monitor uncertainties.
Mean			*3691*	*34*				
**75035**	Rb-Sr	x	3818	127	1.4	[1]	[14]	
	39Ar/40Ar	x	3734	50	1.5	[2]	[15]	Whole rock analysis
	39Ar/40Ar	x	3739	40	9	[2]	[16]	Plagioclase
	39Ar/40Ar	x	3741	40	3.5	[2]	[16]	Plagioclase
Mean			*3741*	*24*				
**71055**	Rb-Sr	x	3632	90	NA	[1]	[17]	

Supplementary Table 1 continued

Data References

[1] Nebel, O., Scherer, E. E. & Mezger, K. Evaluation of the 87Rb decay constant by age comparison against the U–Pb system. *Earth and Planetary Science Letters* **301**, 1–8 (2011).
[2] Renne, P. R., Balco, G., Ludwig, K. R., Mundil, R. & Min, K. Response to the comment by W.H. Schwarz et al. on "Joint determination of 40K decay constants and 40Ar∗/40K for the Fish Canyon sanidine standard, and improved accuracy for 40Ar/39Ar geochronology" by P.R. Renne et al. (2010). *Geochimica et Cosmochimica Acta* **75**, 5097–5100 (2011).
[3] Papanastassiou, D. A. & Wasserburg, G. J. Lunar chronology and evolution from RbSr studies of Apollo 11 and 12 samples. *Earth and Planetary Science Letters* **11**, 37–62 (1971a).
[4] Cliff, R. A., Lee-Hu, C. & Wetherill, G. W. Rb-Sr and U, Th-Pb measurements on Apollo 12 materials. *Lunar and Planetary Science Conference Proceedings* **2**, 1493 (1971).
[5] Compston, W., Berry, H., Vernon, M. J., Chappell, B. W. & Kaye, M. J. Rubidium-strontium chronology and chemistry of lunar material from the Ocean of Storms. *Lunar and Planetary Science Conference Proceedings* **2**, 1471 (1971).
[6] Horn, P., Kirsten, T. & Jessberger, E.K. Are there a 12 mare basalts younger than 3.1 b.y. Unsuccessful search for a 12 mare basalts with crystallization ages below 3.1 b.y. *Meteoritics* **10**, 417–418 (1975).
[7] Eugster, O., Eberhardt, P., Geiss, J., Grögler, N. & Schwaller, H. Cosmic ray exposure histories and 235U-136Xe dating of Apollo 11, Apollo 12, and Apollo 17 mare basalts. *Journal of Geophysical Research: Solid Earth* **89**, C171–C181 (1984).
[8] Papanastassiou, D. A. & Wasserburg, G. J. RbSr ages of igneous rocks from the Apollo 14 mission and the age of the Fra Mauro formation. *Earth and Planetary Science Letters* **12**, 36–48 (1971b).
[9] Stettler, A., Eberhardt, P., Geiss, J., Grögler, N. & Maurer, P. Ar39-Ar40 ages and Ar37-Ar38 exposure ages of lunar rocks. *Lunar and Planetary Science Conference Proceedings* **4**, 1865 (1973).
[10] Borg, L. E., Connelly, J. N., Boyet, M. & Carlson, R. W. Chronological evidence that the Moon is either young or did not have a global magma ocean. *Nature* **477**, 70–72 (2011).
[11] Eugster, O. Chronology of dimict breccias and the age of South Ray crater at the Apollo 16 site. *Meteoritics & Planetary Science* **34**, 385–391 (1999).
[12] Nyquist, L. E., Bansal, B. M., Wiesmann, H. & Jahn, B. M. Taurus-Littrow Chronology: Implications for Early Lunar Crustal Development. *Lunar and Planetary Science Conference Proceedings* **5**, 565 (1974).
[13] Evensen, N. M., Murthy, V. R. & Coscio, M. R., Jr. Rb-Sr ages of some mare basalts and the isotopic and trace element systematics in lunar fines. *Lunar and Planetary Science Conference Proceedings* **4**, 1707 (1973).
[14] Murthy, V. R. & Coscio, M. R., Jr. Rb-Sr ages and isotopic systematics of some Serenitatis mare basalts. *Lunar and Planetary Science Conference Proceedings* **2**, 1529–1544 (1976).
[15] Turner, G. & Cadogan, P. H. Possible effects of 39Ar recoil in 40Ar-39Ar dating. *Lunar and Planetary Science Conference Proceedings* **2**, 1601–1615 (1974).
[16] Turner, G. & Cadogan, P. H. The history of lunar bombardment inferred from 40Ar-39Ar dating of highland rocks. *Lunar and Planetary Science Conference Proceedings* **2**, 1509–1538 (1975).
[17] Tera, F., Papanastassiou, D. A. & Wasserburg, G. J. The Lunar Time Scale and A Summary of Isotopic Evidence For A Terminal Lunar Cataclysm. *Lunar and Planetary Science Conference Proceedings* **5**, 792 (1974).
